# The Continuing Threat of Methicillin-Resistant *Staphylococcus aureus*

**DOI:** 10.3390/antibiotics8020052

**Published:** 2019-05-02

**Authors:** Márió Gajdács

**Affiliations:** Department of Pharmacodynamics and Biopharmacy, Faculty of Pharmacy, University of Szeged, 6720 Szeged, Hungary; gajdacs.mario@pharm.u-szeged.hu; Tel.: +36-62-341-330

**Keywords:** *Staphylococcus*, MRSA, *SSCmec*, colonization, typing, lipoglycopeptides, oxazolidinones, ceftaroline, daptomycin, pleuromutilin

## Abstract

*Staphylococcus aureus* has been an exceptionally successful pathogen, which is still relevant in modern age-medicine due to its adaptability and tenacity. This bacterium may be a causative agent in a plethora of infections, owing to its abundance (in the environment and in the normal flora) and the variety of virulence factors that it possesses. Methicillin-resistant *S. aureus* (MRSA) strains—first described in 1961—are characterized by an altered penicillin-binding protein (PBP2a/c) and resistance to all penicillins, cephalosporins, and carbapenems, which makes the β-lactam armamentarium clinically ineffective. The acquisition of additional resistance determinants further complicates their eradication; therefore, MRSA can be considered as the first representative of multidrug-resistant bacteria. Based on 230 references, the aim of this review is to recap the history, the emergence, and clinical features of various MRSA infections (hospital-, community-, and livestock-associated), and to summarize the current advances regarding MRSA screening, typing, and therapeutic options (including lipoglycopeptides, oxazolidinones, anti-MRSA cephalosporins, novel pleuromutilin-, tetracycline- and quinolone-derivatives, daptomycin, fusidic acid, in addition to drug candidates in the development phase), both for an audience of clinical microbiologists and infectious disease specialists.

## 1. Introduction

Taxonomically, the genus Staphylococcus is included in the Micrococcaceae family within the phylum Actinobacteria [1,2]. They are Gram-positive, catalase-positive, and bacitracin-resistant cocci [3]. Staphylococci are non-spore forming bacteria, nevertheless they are very common in nature and they can survive in a variety of harsh environments outside of the body, in addition to being resistant to many disinfecting agents [1,2,4,5]. *Staphylococcus aureus* is coagulase-positive, which is another important differentiating factor between this species and coagulase-negative staphylococci (CoNS; e.g., *S. epidermidis*) [1,6]. *S. aureus* (and its methicillin-resistant counterpart) may colonize various mucosal sites of the body: the nostrils (nares), throat, dedicated areas of the skin (including the axilla, groin, and perineum; these skin surfaces are usually moist), and rectum. *S. aureus* is an exceptionally successful pathogen, which is still relevant and dangerous in modern age-medicine [7]. Furthermore, small-colony variants of *S. aureus* (SCVs; a sub-population of bacteria that are naturally present in small quantities) allow for chronic, recurrent, and antibiotic-resistant infections to develop and persist in the host [4,8]. In this morphotype of *S. aureus*, mutations occur in the genes that regulate metabolic activity, resulting in the so-called “dwarf colony” phenotype on agar plates, while, *in vivo*, these bacteria can withstand otherwise lethal doses of antibiotics [9,10]. *S. aureus* is an important causative agent of bacteremia and, though hematogenic dissemination, additional infections, such as infective endocarditis, complicated skin and soft tissue infections (cSSTI), osteoarticular infections, prosthetic device infections (PDI), and pleuropulmonary infections may also occur. In addition, *S. aureus* has significant roles in other pathologies (epidural abscesses, meningitis, toxic shock syndrome (TSS), urinary tract infections (UTIs), septic thrombophlebitis, etc.) [5,7,9,11,12,13]. Invasive infections that are caused by this pathogen affect all age groups, while the prevalence of these infections is somewhat higher in infants and patients over 65 years of age [5,12,14,15,16].

The main virulence factors of these bacteria play different functions in various stages of their replication; e.g., in the exponential-growth phase, surface proteins, such as Protein A, elastin-binding protein, collagen-binding protein, fibronectin-binding protein, and clumping factor play major roles [17,18,19,20,21]. In contrast, during the stationary phase, secreted proteins, such as enterotoxin B, toxic shock syndrome toxin-1 (TSST-1), and α-toxin are the most prevalent [17,18,19,20,21]. Various regulatory pathway mediate the expression of these virulence factors, mainly by the *agr* (accessory gene regulator) system [22]. This regulatory system responds to the density of the bacterial populations (this phenomenon is termed quorum sensing (QS)) [23]. At the onset of infection (where rapidly-multiplying bacteria are present, and the density of the population is low), the expression of surface-bound adhesins is more pronounced, while, if high population density is locally achieved (in the stationary phase, generally at the site of infection), the secretion of bacterial toxins commences [5,17,21,22,24].

Before the advent of antibiotics, severe infections with *S. aureus* were usually considered to be a death sentence [25]. These infections became successfully treatable after the paradigm-altering discovery of penicillin by Alexander Fleming, due to the uniform susceptibility of these bacteria [26]. However, this did not last, as only few years after the introduction of penicillin, the first resistant strains were described (nowadays, more than 95% of *S. aureus* isolates are resistant to penicillin), producing an extracellular enzyme, called penicillinase [27,28]. Reacting to the resistance trends, pharmaceutical companies developed methicillin, which may be considered the prototype of anti-staphylococcal penicillins (a group currently consisting of oxacillin, cloxacillin, dicloxacillin, nafcillin (these drugs are available in the US) and flucloxacillin [available in some parts of Europe and Australia]) [29,30]. Shortly, strains also developed a resistance mechanism against these agents, which was unrelated to the production of penicillinases. The phenomenon of methicillin-resistance in *S. aureus* (MRSA) strains was first described in 1961, and they were characterized by an altered penicillin-binding protein (PBP2a, see Section 3.), which had reduced affinity for methicillin and, thus, could continue peptidoglycan synthesis uninterrupted in the presence of this drug [31,32,33]. 

However, this, had more detrimental ramifications than resistance to “just” one antibiotic: MRSA strains show resistance to all penicillins, cephalosporins, and carbapenems, making the β-lactam armamentarium clinically ineffective [33]. This was especially problematic in sensitive age groups (during pregnancy and in children), as many other antibiotics are not suitable to be used due to their teratogenicity or their severe side effects [12,34]. Furthermore, over time, MRSA strains became resistant to a number of other antibiotic classes (e.g., fluoroquinolones, macrolides, aminoglycosides, clindamycin). For this reason, MRSA strains, coupled with other resistance mechanisms, may be considered as the first class multidrug resistant (MDR) pathogens [35,36,37]. Nowadays, targeted antibiotic therapy, which is aided by antimicrobial susceptibility testing results (as a part of antibiotic stewardship), is of critical importance. In addition, the preservation and prudent use of these drugs is a clear agenda, both from the healthcare and regulatory perspectives. There is a debate on whether he imprudent use of antibiotics at that time catalysed the appearance of MRSA, or whether the development of penicillin-resistance to methicillin-resistance was a clear evolutionary path, which was irrespective of drug utilization levels [38,39,40,41,42,43,44]. Unsurprisingly, studies comparing hospital costs, the length of hospital stay, and mortality rate related to methicillin-susceptible and resistant *S. aureus* infections clearly highlight that MRSA infections are associated with a greater burden on healthcare infrastructure [45,46]. Delayed therapy may increase the risk for the development of MRSA bacteremia: despite the availability of active antibiotics for the treatment of these infections (see Section 4.), it is still a major cause of morbidity and mortality worldwide [47,48,49,50].

Between the 1960–1970s, MRSA infections were predominantly associated with nosocomial outbreaks, which affect hospitalized patients or outpatients that frequently attend hospitals [51]. These infections, termed *hospital-acquired MRSA* (HA-MRSA) were in majority until the end of the 1970’s. The first successful clone, whose global spread was described, was the phage type 83A (sequence type 250 [ST250]; see Section 3.), which was gradually replaced by other clones during the 1980s [52,53]. The epidemiology of MRSA infections shifted during the 1990s, when the number of infections, lacking the risk factions that are associated with acquiring HA-MRSA increased significantly [54]. This has led to the emergence of community-associated MRSA (CA-MRSA) infections, including infections that were diagnosed in outpatients or inpatients within two days of hospitalization, not being associated with previous healthcare-related risks (long-term care facilities, hospitalization in the previous year, surgical procedures, hemodialysis, presence of an indwelling catheter, or a percutaneous device) or a previous isolation of MRSA from the same patient (see CDC-defined case-definitions of CA-MRSA) [55,56]. The spread of CA-MRSA has been reported from basically every region on the planet and this has become the principle type of MRSA infection in the past 10–20 year, owing to successful clones [57,58]. In both MRSA-subtypes, the emergence of additional resistance mechanisms (both due to mutations and acquired resistance determinants) has been a constant feature [31,40]. From the 1980s and onward, several antibiotics appeared on the market, allowing for the better management of infections, nonetheless, the emergence and spread of resistance to these drugs has been observed throughout the years [16]. For example, the resistance against fluoroquinolones has increased drastically not long after the introduction of ofloxacin and ciprofloxacin [59,60]. In fact, fluoroquinolone-resistance was considered as a hallmark of HA-MRSA bacteria, thus, it was used as a method of differentiation among the MRSA strains of nosocomial and community origin [55,61]. The differentiation between HA- and CA-MRSA is further complicated by the phenomena of the so-called community-onset MRSA (CO-MRSA) infections: these infections are thought to be related to HA-MRSA infections (they are also called “escaped” or “feral” MRSA strains, because they have escaped from the nosocomial environment), which are associated with the increasing use of outpatient intravenous (parenteral) antimicrobial therapy (OPAT) and the management of complex infections in the home of the patients [55,62,63].

*S. aureus* is also a significant cause of morbidity and mortality in animals. This is especially important for the livestock/food industry, as outbreaks may result in pronounced economic losses [64]. Livestock-associated MRSA (LA-MRSA) strains were first described in the beginning of the 2000s in livestock; today, it is well-known that veterinary doctors, people working on farms or at slaughterhouses, or basically anyone who comes in contact with any animal or pet, which is carrying LA-MRSA is at risk of transmission [65]. LA-MRSA has no relevant host-specificity, it can colonize any animal, although cattle, pigs, and poultry are reported to be the main reservoirs [65,66,67]. LA-MRSA initially presented itself as a conundrum to scientists, as they were non-typable with pulsed-field gel electrophoresis (PFGE; after digestion with the *SmaI* restriction enzyme) at the time of their discovery. After additional studies, it was revealed that this new lineage of *S. aureus* belongs to clonal complex 398 (CC398) [68]. No difference in the pathogenicity of LA-MRSA—compared to the nosocomial- or community-acquired types—was found [65,69]. The epidemiological data is scarce regarding the prevalence of LA-MRSA infections, some reports suggest that they account for approximately 15% of MRSA SSTI in the community and 1–2% of infections that were isolated in the hospital environment [70].

The landscape of nosocomial and MDR pathogens in clinical practice has drastically changed since the beginning of the 21st century (i.e., the global spread of toxin-producing *Clostridium difficile* [71,72,73], multidrug-resistant *Neisseria gonorrhoeae* [74], and the concerning rise of extended-spectrum β-lactamase (ESBL)- and carbapenemase-producing *Enterobacteriaceae* [75,76]); however, MRSA has proven to be one of the most persistent drug resistant pathogens in both the healthcare and community setting [56]. Based on the assessments of the US Centers for Disease Control and Prevention (CDC), after considering various factors, such as treatability, mortality, burden on the healthcare-infrastructure and the community, prevalence and increasing trends of resistance, preventability and transmissibility, in addition to the drugs that are currently in the pipeline, MRSA has been classified as a *serious threat* [77]. *S. aureus* is included in the group of “*ESKAPE*” bacteria, which comprise the MDR pathogens that are currently considered as the biggest concern for humanity [35,40,78]. Although there is a relative abundance of the different antibiotic-groups for the treatment of MRSA (this is one reason why pharmaceutical companies became more focused on the development of new drugs against MDR Gram-negatives in the 21st century, see Section 4.) [79], one should not be complacent about the current situation [80,81,82]. This is underlined by the recent WHO report, urging drug companies to invest and target various drug-resistant bacteria (causing serious morbidity and mortality worldwide) during antibiotics research, which also includes MRSA [83,84].

## 2. MRSA Colonization and Screening

In the current climate of the antibiotic-resistance crisis, it is important for laboratories to monitor the trends and mechanisms of resistance in *S. aureus* (especially methicillin/oxacillin-resistance), in addition to the spread of successful clones [85]. The detection of carriage is another important hallmark in infection control and the successful eradication of MRSA, which is among the responsibilities of clinical microbiology/public health laboratories [86]. This is further highlighted by the fact that, in most cases, colonization (lasting for periods of few months to a few years) precedes infection [87]. Colonization with MRSA is a well-known risk factor in developing an MRSA infection in adults and children; this is especially true for patients who acquire MRSA colonization in the nosocomial setting, where the risk of developing an MRSA infection as a result is around 30% [85,88]. MRSA is usually spread by direct skin-to-skin contact and this may occur during hospital admission, transfer, or other healthcare-related contact; however, the role of shared public spaces (e.g., dormitories, gym, barracks, etc.) was also noted [86]. A set of bacterial determinants influence carriage (adhesive proteins (SdrC, SdrD, and SdrE), clumping factor, fibronectin binding proteins (clfA, clfB, fnbA, fnbB), adhesive molecules (altA, eap), cell surface-remodeling enzymes (sceD, oatA, altA), and biofilm formation) and host-specific factors (the integrity of the skin barrier, chronic inflammation, toll-like receptor 2 (TLR-2) gene polymorphisms, antimicrobial peptides (AMPs), such as cathelicidins, defensins, proteolysis-inducing factor, lactoferrin, RNase 7; individual variation in cortisol and 25-OH-D-vitamin levels, HLA-DR3 allele) [86,88]. Based on the patterns of carriage, persistent, intermittent, and non-carriage has been described. The so-called “culture rule” was established for the appropriate detection of these carriage types: based on two nasal cultures taken a week apart, the number of positive cultures (two: persistent, one: intermittent and zero: non-carrier) was indicative of carriage status [89]. Screening should be performed from multiple sites of the body to ensure the adequate pickup rate of this pathogen. Additionally, it would be ideal to simultaneously process these samples for financial considerations [90].

**Figure 1:**
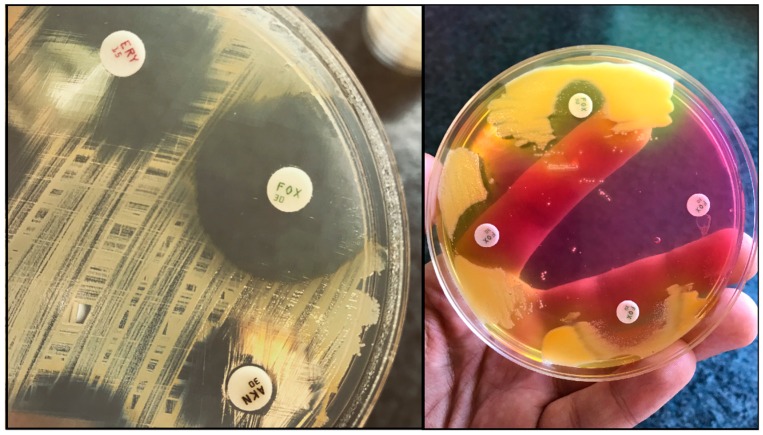
Cefoxitin-susceptible *S. aureus* on Mueller-Hinton-agar (antibiotic susceptibility-testing based on the Kirby-Bauer method) (**left**), Methicillin-resistant *S. aureus* (MRSA) screening using mannitol-salt agar (MSA) using cefoxitin disks (**right**).

The most frequently used methods for MRSA screening are still culture-based [91]. Generally, all of these methods include a preliminary step of selective enrichment in a broth medium, followed by culturing on selective solid media (containing oxacillin/cefoxitin) (Figure 1.) [92]. Several studies have highlighted the relevance of enrichment, showing that the direct plating of the sample onto MRSA-selective agars has inadequate selectivity and sensitivity [93]. Instead of methicillin (nowadays only having theoretical importance) or oxacillin (the use of which is not recommended anymore, because it is affected by other resistance mechanisms that are related to β-lactams), the use of cefoxitin disks, as a surrogate agent, is recommended, both by the Clinical Laboratory Standards Institute (CLSI) and European Committee on Antimicrobial Susceptibility Testing (EUCAST) guidelines [94,95,96]. Clearly, any anomaly that was detected around the cefoxitin (<22 mm zone diameter; together with the knowledge of local epidemiological data) during routine antimicrobial susceptibility testing (AST) should be investigated if the patient’s MRSA carriage is unknown [94,95,96]. Around 48 hours are needed for identification (considering enrichment and growth on selective solid media) and the reporting of results using culture-based methods [91]. If cefoxitin susceptibility testing is not performed in parallel, another day may be required to perform AST. Alternative methods, such as latex agglutination, can also be used: these tests detect the product of the *mecA* gene (the PBP2a protein, found in the cell membrane of MRSA). The disadvantage of these methods is that the product of the *mecC* gene (see Section 3.) is not detected, which may lead to false-negative results. Additionally, selective enrichment prior to detection with latex agglutination is still recommended.

With the advent of molecular methods (polymerase chain reaction, PCR), the reporting of results on the same day has become possible, although this means that the samples must reach the laboratory in time for batch processing [91]. These methods may include in-house PCRs (with the design of target-specific primers for the relevant genes) or commercially available systems (e.g., the Cepheid Xpert MRSA assay) [91,97]. Interestingly, several reports have also indicated the usefulness of matrix-assisted laser desorption-ionization time-of-flight mass spectrometry (MALDI-TOF MS) in the detection of MRSA, together with the selective enrichment and/or PBP2a’ latex agglutination, which reduces the turnaround-time (TAT) for result reporting [92,98]. Appropriate logistics and infrastructure are needed to support the effectiveness of MRSA screening: including swift transport of the specimens from the ward to the microbiology laboratory, reporting of results to the clinicians in a clinically-relevant timeframe, and prompting action from the part of the infection control unit (including isolation and/or decolonization of the patient) [91,97]. In summary, the methods that are used for the detection of MRSA-colonization are usually determined by the settings of the healthcare institution (i.e., number of beds and patient characteristics) and the facilities of the clinical microbiology laboratory (i.e., the expected sample number), the required TAT for detection and monetary constraints [91,92,97]. Currently, there is no study confirming that the cost/benefit ratio and the overall benefits of PCR screening could surpass the culture-based techniques. Besides clinical microbiology laboratories, the abovementioned techniques are also used in the MRSA screening in livestock, based on the same principles. However, for national-level surveillance purposes, or during the investigation and follow-up of an outbreak, molecular methods (PCR) are the most frequently used, being complemented with genotyping (see Section 3.).

## 3. Genetics of MRSA, Typing Methods

By definition, the MRSA strains are *S. aureus* isolates that possess an altered penicillin-binding protein (namely PBP2a or PBP2c, encoded by the *mecA/mecC* genes; the expression of which is regulated by *mecI* and *mecR1*), a protein that is essential to bacterial cell wall synthesis, resulting in non-susceptibility to all β-lactam antibiotics, with the exception of anti-MRSA cephalosporins (ceftaroline and ceftobiprole) [53,99]. The resistance that is caused by modifications in the PBPs is not a unique phenomenon to *S. aureus*, as similar mechanisms are responsible for ampicillin-resistance in *Enterococcus faecium* (PBP5) [100] and the penicillin non-susceptibility in *Streptococcus pneumoniae* (PBP1x mosaic genes) [101]. In addition, these microorganisms can easily acquire resistance to additional antibiotic groups due to horizontal gene transfer [102]. The genes that encode for these proteins can be found in a chromosomal genetic element, called staphylococcal chromosomal cassette *mec* (*SCCmec*), which was found as a fundamental discovery by Hiramatsu et al. [103]. *SCCmec* is integrated into *orfX* (a staphylococcal gene of unknown function) and in addition to the *mecA* or *mecC* complex (which is a 30–60 kbp genetic element), it contains two recombinase genes (*ccrA* and *ccrB*), and is responsible for the integration/excision of this genetic element from the staphylococcal chromosome [104].

Currently, twelve allotypes of *SCCmec* (namely I–XII) have been defined, which are separated based on the type of *ccr* gene complex (responsible for site-specific excision and the insertion of the gene cassette) and the type of *mec* complex [102,105]. Presumably, this number is only going to increase (with more subtypes emerging), because the use of novel sequencing technologies (i.e., next-generation sequencing, NGS) with higher discriminatory power will also undoubtedly bring forth changes in this field, just like after the first time the *S. aureus* genome was first sequenced in 2001 [106,107]. In the 1980–1990s, CA- and HA-MRSA strains could be safely distinguished by phenotypic characteristics: the HA-MRSA strains were more frequently MDR strains (resistant to antibiotics other than β-lactams), while the CA-MRSA strains were predominantly susceptible to non-β-lactams [31]. However, this distinction between the two groups has eroded slowly over time [108]. Nowadays, a useful method for the differentiation of CA- and HA-MRSA strains is based on molecular methods. When compared to the community-associated strains, HA-MRSAs carry a larger *SCCmec* cassette and they usually belong to the I, II, or III allotypes [51,85,102]. In contrast, the CA-MRSA strains are associated with smaller genetic elements (carrying the *mecA/C* gene), which is thought to be influencing their mobility, belonging to the IV, V, or VII allotypes [57,61,85,102]. The detection of Panton–Valentine leukocidin (PVL) is another possible way for distinction, which is a characteristic toxin of the species [17,108,109]. Although the clinical role and significance of this toxin in the pathogenesis of the diseases that is caused by *S. aureus* is not clear, the presence of the toxin is much more frequent in the CA-MRSA strains [109,110,111].

When compared to *mecA*, *mecC* is a novel variant of the gene responsible for methicillin-resistance (encoded on a novel type XI SCC*mec* element [99]), which was first described in 2007 (although retrospective studies with old strain collections have found that these variants were probably around, ever since MRSAs were known) [112]. *mecC* shows around 70% nucleotide sequence homology with the classical *mecA* gene, resulting in false negative results in molecular detection systems [113]. Following 2010, *mecC*-positive MRSA strains are increasingly being reported in both humans and animal infections [113,114]. In fact, some studies suggest a zoonotic background and transmission for this allotype. There is limited data in the literature regarding the efficacy of detecting *mecC*-positive strains, using classical (i.e., phenotypic) AST methods. Another reason for the use of cefoxitin as a surrogate for MRSA-detection is that it was found to be more reliable in the detection of *mecC*-positive MRSAs (see Section 2.) [96]. Owing to the genetic nature of the *SCCmec* cassette (being a mobile genetic element), methicillin-resistant CoNS may also possess the *mecA/mecC* genes, which may result in dual colonization with methicillin-resistant CoNS and methicillin-susceptible *S. aureus* [112].

Beginning from the 1980s (after the global spread of some successful MRSA clones), the need for the observation and characterization of the epidemic MRSA clones has become of pivotal importance [85]. Five different methods, with various advantages and disadvantages associated with each method, currently perform the typing of successful epidemic clones. Initially, phage typing was used for the differentiation of various MRSA strains, owing to their differential susceptibility of lytic phages (differentiating strains into phage types) [52]. Multi-locus sequence typing (MLST) is sequence based genotyping method, which is based on the single nucleotide polymorphisms of seven distinct *S. aureus* housekeeping genes (covering around 450 bps of genetic material) [115]. Using the allelic profile of various strains, a sequence type (e.g., ST44) can be assigned. If strains have identity at ≥5 housekeeping genes, they are assigned to the same clonal complex (e.g., CC78). Nowadays, the lineages of various epidemic strains are considered based on the CC identity [99]. The advantage of MLST is the ability to monitor variations over a longer time period to follow the evolution of the epidemic clones and its usefulness in scientific analysis; however, it may be too expensive for routine laboratories [85]. Pulse-field gel electrophoresis (PFGE) is more suitable for the evaluation of rapid changes over a shorter time period [116]. During this procedure, *S. aureus* genomic DNA is digested by *SmaI* restriction enzyme and the resulting fragments are then separated by pulsed-field electrophoresis in an agarose gel. The clustering of strains is performed based on >80% similarity, separating clones into various PFGE types (e.g., C1, D5, G10) [85,116]. Several national and international PFGE databases are available for the comparative analysis of epidemic MRSA strains [1,116,117]. Protein A typing (*spa* typing) is an inexpensive method, which is based on the sequence analysis of variable number tandem repeats (VNTRs) in the encoding gene of this protein (*spa*), while also taking the number of repeat variations and point mutations into account (e.g., t011, t899) [118,119,120]. As a novelty, some studies have reported in the use of MALDI-TOF MS for the rapid discrimination of epidemic clones of MRSA, based on the association of their measured protein peaks (for an excellent review on the background of mass spectrometry, see [121]) and their *spa* types [118,122]. Whole-genome sequencing (WGS) is currently considered to be the gold standard as a molecular typing tool for the investigation of MRSA outbreaks [123]. This method has the most discriminating profile, allowing for the analysis of core genome multi-locus sequence typing (cgMLST) data [106]. The price of the sequencing machines and the lack of the adequate bioinformatics pipeline hinders the extensive use of this method, therefore sequencing is usually limited to reference laboratories [106,123].

## 4. Treatment Considerations, Emerging Concepts 

Table 1 presents the currently available drugs for the therapy of MRSA-infections. Following the emergence of MRSA in clinical practice, the significance of non-β-lactam-antibiotics (notably trimethoprim-sulfametoxazole [SMX/TMP] and doxycycline) has increased, especially in the therapy of cSSTI that is caused by CA-MRSA infections [45,124,125]. However, acquired resistance against the abovementioned drugs developed rapidly. At the end of the 1980s, MRSA strains with resistance against all other drugs except vancomycin were very common [108]. Resistance against SMX/TMP may occur due to alternative metabolic pathways of folate synthesis (due to point mutations in the *dhfr* gene) [126,127], while various *tet* efflux pumps and target modification (30S ribosomal RNA) mainly affected doxycycline [128]. Resistance to fluoroquinolones also frequently occurs in MRSA and is due to mutations in the quinolone-resistance-determining region (QRDR) of DNA gyrase (*gyrA* and *gyrB*) and topoisomerase IV (*grlA* and *grlB* in *S. aureus*) [59,60]. In addition, the overexpression of the Major Facilitator Superfamily (MFS) efflux pumps NorA and NorB contributes to high-level fluoroquinolone resistance (not to mention, the resistance to several antiseptics and disinfectants) in *S. aureus* [129,130,131,132].

For this reason, vancomycin (dosing generally includes a 25–30 mg/kg loading dose, followed by 15–20 mg/kg maintenance dose) was the “gold standard” of anti-MRSA-therapy for a very long time [133,134,135]. It exerts potent bactericidal activity in a concentration- and time-dependent manner against Gram-positive bacteria (including *Flavobacterium* spp. (which are Gram-negative) and excluding *Erysipelotrix* spp., *Lactobacillus* spp., *Leuconostoc* spp., and *Nocardia* spp. (due to intrinsic resistance to the drug)) [133,134,135]. However, the use of vancomycin also had pronounced drawbacks: it could only be parenterally used, as it is not absorbed in the gastrointestinal tract (which may even be useful in some cases; see therapy of *C. difficile* infections), its side effect profile (nephrotoxicity, ototoxicity, red man syndrome due to histamine liberation, etc.), and the subsequent need for therapeutic drug monitoring (TDM) [135,136]. The biggest concern is the phenotype of vancomycin-resistant *S. aureus* (VRSA; MIC ≥ 16 μg/mL), which has acquired the *vanA* gene from vancomycin-resistant *Enterococcus* spp. (where the prevalence of this gene is much higher) [100,137,138], while the vancomycin intermediate-resistant *S. aureus* (VISA; MIC, 4–8 μg/mL) strains are characterized by reduced susceptibility, owing to a thickened cell wall, which is capable of binding the drug and reducing its diffusion into the cell [139]. Resistance against teicoplanin characterizes a similar phenotype (Targocid^®^ [US/EU]; another member of the *glycopeptide* group of antibiotics; 800 mg/12 h), but not vancomycin, which is caused by the presence of the *vanB* gene [140]. It is important to note that the step-wise progression of VISA-to-VRSA does not occur, as the two resistance types have completely different mechanisms [139]. Luckily, the prevalence of VRSA remains very low, and most published reports detected strains that were colonizers, not the causative agents of infection [137,138]. An additional phenotypic group that warrants attention is the heterogeneous vancomycin intermediate-resistant *S. aureus* (hVISA; 1–4 μg/mL): these bacteria are described as being at a stage prior to the development of intermediate-level resistance [141]. With continuous selection pressure from vancomycin treatment, the environment favors the selection of VISA clones, which leads to a unanimous intermediate-resistant population. Based on the available experimental evidence, multiple, sequential mutations (involving various regulatory systems of cell wall homeostasis and remodeling) are required [141]. The role and clinical significance of hVISA is not yet understood, as there are no standardized methods to appropriately study this phenomenon in clinical microbiology laboratories [142].

Three novel antibiotics from the *lipoglycopeptide family* have been approved in the period between 2009–2014 by the US Food and Drug Administration (FDA) for the therapy of infections that are caused by (MDR) Gram-positive bacteria: telavancin (Vibativ^®^ [US]; 10 mg/kg over 8 h), dalbavancin (Dalvance^®^ [US], Xydalba^®^ [EU]; 1500 mg/single dose) and oritavancin (Orbactiv^®^ [US/EU]; 1200 mg/single dose) [133,143]. Telavancin and oritavancin are semisynthetic derivatives of vancomycin with a hydrophobic side chain that is attached to the vancosamine sugar, while dalbavancin is a derivative of teicoplanin [133,143]. These drugs exhibit concentration-dependent antibacterial activity. They all show activity against hVISA/VISA, while oritavancin also exhibits antibacterial activity against *vanA*-positive *S. aureus* and *Enterococcus* spp. [144]. They are approved for complicated skin and skin structure infections (cSSTI), bone and joint infections, hospital-acquired (HAP), and ventilator-associated bacterial pneumonia (VAP) [133,143]. Their antimicrobial activity is attributed to a double mechanism: inhibition of cell wall synthesis (as seen previously in vancomycin) and the disruption of the integrity cell membrane barrier, which leads to permeabilization and cell death [133,143]. The antibacterial activity of these drugs is due to the novel combined action of inhibition of the cell wall synthesis and the disruption of bacterial cell membrane barrier function [145]. The additional advantage of dalbavancin and oritavancin in their long half-life (>300 h), therefore their half-life, coupled with their concentration-dependent activity, is appropriate for single-dose (once weekly) treatments, especially in OPAT settings. They do not require a loading dose or TDM [133,143]. However, when compared to vancomycin, they are much more expensive, which currently hinders their more widespread use in clinical practice. Another potential disadvantage of these drugs is that, due to their high levels of plasma protein binding (PPB > 90%), they are not removed by conventional dialysis. An increase in the mortality rate in patients that were treated with renal insufficiency was also described [79,133,143].

The class of oxazolidinones is another important antibiotic group in the treatment of drug resistant Gram-positive infections. Oxazolidinones were first described in the 1970s, while the first drug linezolid was approved in 2000 by the FDA. Oxazolidinones were perceived as attractive drugs due to several features; firstly, the novel mechanism of action (inhibition of protein synthesis by binding to the peptidyl transferase A-site of the 50S subunit of ribosomal RNA) [146]; secondly, their spectrum of activity Gram-positives, including MDR strains), the option for intravenous-to-oral switch with excellent oral bioavailability, and the lack of cross-resistance with different antibiotics [147]. Linezolid (Zyvox^®^ [US], Zyvoxid^®^ [EU]; 600 mg/12 h) and tedizolid (Sivextro^®^ [US/EU]; 200 mg/24 h) was approved for the treatment of nosocomial and community-acquired pneumonia, complicated and non-complicated skin, and soft tissue infections (including MRSA) and bone and joint infections, while they are not recommended for the treatment of bacteremia and endocarditis. Tedizolid is a prodrug (phosphate), which is hydrolyzed by plasma or intestinal enzymes in *vivo* to produce the active form of the drug [147]. Tedizolid (the second-generation oxazolidinone) has more potent antibacterial activity, a longer half-life (12 h), and less pronounced toxicity. Due to its higher (75–80%) protein binding affinity, lower doses should to be administered (cf. linezolid). They have high *in vitro* activity against MRSA; however, there are few data regarding its efficacy against hVISA/VISA and vancomycin-resistant *S. aureus* (VRSA). The drawbacks of this antibiotic group are related to their pharmacodynamic properties: a. drug-drug interactions, which may have severe consequences (e.g., due to synergism with sympathomimetic drugs (various α- and β-receptor agonists), their co-administration may result in an acute hypertensive episode; they increase the toxicity of opioid analgesics and fibrates (drugs against hyperlipidemia); they may also increase the pharmacological effects of insulin and oral antidiabetic drugs (like metformin and glimepiride)), b. serious side effects, such as serotonin-syndrome (because they can inhibit the monoamine-oxidase-A (MAO-A) enzyme) and myelosuppression (which may lead to hematological issues) [147,148]. Their clinical role in the treatment of VRSA infections is unknown due to the lack of data. The prevalence of oxazolidinone resistance is very low and causative mechanism includes changes in L3/L4 ribosomal proteins and methylation of the 23S rRNA, encoded by the *cfr* gene [149]. Bacteria carry multiple copies of the genes encoding 23S rRNA; therefore, for a pronounced increase in the MIC, the mutation needs to be present in several copies (“gene-dose” effect) [148]. These mutations frequently occur in CoNS strains. The carriage of *cfr* does not affect tedizolid (approved in 2014) susceptibility. Although the endemic areas have been reported, the carriage of *cfr* genes continue to be very rare, even though it is a transmissible determinant of (presenting with a risk of developing MDR/XDR infections) [149]. However, some reports suggest that increasing the dose of the antibiotic could overcome *cfr*-mediated resistance (unlike mutational resistance) [148,149]. Clinical trials are currently underway regarding contezolid (MRX-1), which is a third-generation oxazolidinone, for the treatment of cSSTI, complicated intra-abdominal infections (cIAI) and cUTIs [82].

Daptomycin (Cubicin^®^ [US/EU]; 6mg/kg over 24hrs) is a lipopeptide antibiotic, which showed pronounced activity against various Gram-positive bacteria, including MRSA [150]. It is bactericidal, damaging the cell membrane and the membrane potential in a calcium-dependent fashion. It has been postulated that the activity of this compound is most pronounced at the division septum of bacteria. Daptomycin is approved for the treatment of skin and skin structure infections, bloodstream infections, and infective endocarditis, while it is contraindicated in pneumonia, because of its interaction with pulmonary surfactant [150,151,152]. This drug is not available for oral use, only as a parenteral formulation. Electrostatic repulsion of the drug molecule is thought to mediate resistance to daptomycin, owing to the increased positive charge of the bacterial cell surface. The gene that is responsible for daptomycin resistant phenotype is *mpfF*, which results in the incorporation of lysine (a positively charged amino acid) in the peptidoglycan layer: this will increase the positive charge of the cell envelope, which inhibits the binding of daptomycin. In addition, genes encoding cardiolipin synthetases (*pgsA*, *cls*), which affect phospholipid metabolism, were also implicated [150,153].

Ceftaroline (Teflaro^®^ [US], Zinforo^®^ [EU]; 600 mg/12 h) and ceftobiprole (Zevtera^®^ [US], Mabelio^®^ [EU]; 500 mg/8 h) are broad-spectrum cephalosporins with pronounced bactericidal activity against MRSA, VISA, daptomycin-resistant *S. aureus,* and vancomycin-resistant *E. faecalis* [154,155,156,157,158,159,160,161]. For this reason, they are sometimes termed anti-MRSA cephalosporins, or fifth generation cephalosporins [49,79]. It is important to note that these drugs are hydrolyzed by ESBL-enzymes, therefore they may not be appropriate for mixed infections involving Gram-positive and Gram-negative pathogens [156]. They have low minimum inhibitory concentrations for MRSA, which corresponds to their high affinity to the PBP2a/c proteins [154,155,156,157,158,159,160]. Ceftaroline and ceftobiprole are both approved for the treatment of cSSTI, CAP, and HAP (excluding ventilator-associated pneumonia), with ceftaroline being additionally approved for MRSA bacteremia [154,155,156,157,158,159,160]. Resistance to ceftaroline (with MIC values ≥4 μg/mL) was published, but the number of cases is very low, and the underlying mechanism is not yet understood [162]. An interesting therapeutic strategy involves the combination of these anti-MRSA cephalosporins and a glycopeptide or daptomycin (which is currently being evaluated in the CAMERA-2 trial) in the treatment of MRSA bacteremia, although this has been criticized for the increase in antibiotic use and therapeutic costs [163].

The role of tetracyclines in the therapy of MRSA infections has faded over the years, due to various mechanisms of acquired resistance [108]. There were high hopes for tigecycline (Tygacyl^®^ [US/EU]; 50 mg/12 h), a broad-spectrum agent and member of *glycylcycline* family of drugs, which was approved in 2005 for the therapy of cSSSI, cIAI, CAP [164,165,166]. Shortly after its approval, tigecycline received “black box” warning from the FDA, due to the significant increase in the mortality of patients that were treated with tigecycline, which is in contrast to the comparator drugs [167]. In addition, difficulties reaching the therapeutic serum levels for bacteremia can be considered to be drawbacks of this drug [164,165,166]. Eravacycline (Xerava^®^ [US]) and omadacycline (approved in the end of 2018; Nuzyra^®^ [US]; 100–150 mg/24, depending on the administration form) are novel drugs of the tetracycline group [168,169,170,171]. They were mainly developed for the treatment of MDR infections, and they are not affected by resistance mechanisms that are associated with tigecycline or other drugs of this family. Chemically, eravacycline is closely related to tigecycline, while omadacycline is an aminomethylcycline-derivative [168,169,170,171]. Omadacycline is licenced for the treatment of CAP and SSTIs. It has a broad spectrum of activity, including many multi-drug resistant strains of bacteria [170,171]. Phase III studies of eravacycline for cIAI and cUTI finished recently, where this drug presented inferiority to levofloxacin [168,169]. During the trials of both drugs, therapy had to be discontinued in some cases, due to severe nausea and vomiting [168,169,170,171]. It should be noted that these drugs do not cover *Pseudomonas aeruginosa* and *Proteae* (*Proteus, Morganella, Providencia*); therefore, they should not be used in mixed infections where these pathogens are suspected.

The clinical efficacy of some novel quinolones (avarofloxacin, finafloxacin, zaborfloxacin, nemonoxacin) is currently being assessed in a plethora of clinical trials for various indications, such as cSSTI, CAP, HAP, cUTI and sexually transmitted infections (mainly MDR gonorrhea), while delafloxacin (Baxdela^®^ [US]; 300 mg/8 h) has already been approved by the FDA for the therapy of cSSTI, with the evaluation of CAP and cUTI trials currently underway [172,173,174,175,176,177,178]. Unlike previous fluoroquinolones (which were zwitterionic), delafloxacin is anionic in character, which results in the accumulation of the drug in the intracellular space of bacteria, in phagocytes, urine, abscesses, and the gastic juice [173]. As these drugs are all representatives of the quinolone drug family (which has been a center of controversy lately, due to their side effect profile [179]), delafloxacin immediately received a “black box” warning by the FDA (for adverse events, such as peripheral neuropathy, tendinitis, *C. difficile* enterocolitis, and the worsening of myasthenia gravis, QTc-prolongation) and it is expected that the other novel drugs will receive similar designation [173]. Novel quinolones are broad-spectrum agents, active against fluoroquinolone (levofloxacin/moxifloxacin)-resistant bacteria, although some cases were reported where elevated delafloxacin MICs were observed [172,173,174,175,176,177,178].

Iclaprim, which is a diaminopyrimidine-type dihydrofolate-reductase (DHFR) inhibitor, is also being investigated for its efficacy against MRSA infections [180]. This drug-candidate was first developed for SSTIs; however, in the beginning 2010s, the FDA rejected further development plans due to QTc prolongation and the failure to show non-inferiority to the comparator drug. Following the subsequent acquiring of the license of iclaprim by another company, new clinical studies are underway. Iclaprim presented non-inferiority to vancomycin in two phase III studies targeting SSTIs, and a new indication for the drug (therapy of *S. aureus* infections in cystic fibrosis patients); nonetheless, the side effect profile, which is associated with prolonged use (liver toxicity), is concerning [127,181]. The dose used in these trials was 80 mg/12 hours. Iclaprim is not affected by the resistance mechanisms, which hindered the use of SMX/TMP previously [45,124,125].

Mupirocin (Bactroban^®^ [US/EU]) is used as a topical agent for the nasal decolonization of MRSA patients; in addition, it received a new indication for the topical therapy of impetigo [88,182]. The mechanism of action is through binding bacterial isoleucyl tRNA synthetase, resulting in the inhibition of protein synthesis. Resistance to mupirocin has been described: low-level resistance is characterized by point mutations in the *ileS* gene, in contrast, high-level resistance is plasmid-mediated (*mupA* gene), which code for a mutant isoleucyl tRNA synthetase, to which mupirocin is unable to bind [88,183].

Fusidic acid is another drug that was mainly used as a topical preparation for the treatment of skin infections (alone or in combination with topical steroids), atopic dermatitis, and in eye drops, in addition to MRSA decolonization (in combination with rifampicin) in the case of mupirocin resistance [49,88]. The safety and efficacy of fusidic acid orally or intravenously (as it is available in both forms) has also been demonstrated in combination with other antibiotics (e.g., vancomycin, gentamycin, levofloxacin) in the therapy of severe staphylococcal/MRSA-infections, typically in complicated SSTIs, osteomyelitis, and septic arthritis, owing to the excellent penetration of this drug into skin blisters, joints, and the bone tissue [184,185]. Unfortunately, resistance frequently emerges against this steroid-based antibiotic, particularly after prolonged therapy; the resistance levels are range between 0.3–64% worldwide, with significant variation in the prevalence of various resistance determinants in different geographical region [49,88]. This phenomenon is especially frequent in the CA-MRSA isolates, being mediated by the mutations in the chromosomal *fusA* (encoding elongation factor G) or *rplF* (or FusE, encoding ribosome protein L6) genes, or the acquision of the transferable *fusB, fusC* or *fusD* genes (protection of drug target site) [184,185]. When it comes to its antimicrobial mechanism of action, fusidic acid is a protein synthesis inhibitor, which interferes with ribosomal translation. Around 90% of the drug binds to the plasma proteins and the elimination half-life is around 8–10 hours [88]. Currently, there are Phase II studies underway evaluating its efficacy in cSSTI and bone-joint infection [184,185].

Pleuromutilin derivatives are well known drugs since the 1950s, with valnemulin and tiamulin being used routinely in veterinary medicine worldwide [186]. These drugs are inhibitors of protein synthesis, acting on the 50S ribosomal subunit (binding to peptidyl-transferase on the 23S rRNA). At the time of their discovery, azamulin was the primary drug candidate, but, due to its pronounced effect on the liver microsomal enzymes (CYP inhibitor), liver toxicity, and the availability of drugs with more advantageous properties, its development was discontinued [186]. Nevertheless, in the current age of MDR pathogens, there is a fresh interest in pleuromutilin-type antimicrobials for human use. Retapamulin (Altrabax^®^, Altargo^®^ [US]) was the first class of these drugs to receive approval from the FDA, and it is currently used as a topical antibiotic (for the treatment of impetigo) [187,188,189]. There is interest in retapamulin to be used in decolonization regimens for MRSA as cross-resistance with mupirocin is unlikely, although clinical trials are needed to assess the value of this drug in the abovementioned indication [190]. Besides retapamilin, azamulin and lefamulin are too in the clinical (Phase II) phase of drug development in SSTIs and CAP that is caused by MDR Gram-positive bacteria [186].

Various emerging therapeutic approaches are currently in development for the treatment of MRSA and other related MDR infections, including antimicrobial peptides (AMPs, such as magainin, pexiganan) [191,192], quorum sensing-inhibitors (or quorum quenchers) [23], inhibitors of virulence factor-expression [193,194,195], efflux pump inhibitors [132,196,197], probiotics [193], repurposed natural compounds with antimicrobial activity (predominantly for topical use) [198,199], phage therapy [200,201], and the development of anti-staphylococcal vaccines [202,203]. There are numerous studies that indicate the promise of combating MRSA infections with AMPs; their mechanism of action mainly consists of creating pores on in the intact bacterial membrane, which leads to cell lysis [191,192,204]. However, there have been many difficulties (e.g., lack of stability *in vivo*, immunogenicity, toxicity to red blood cells, enzymatic degradation) in their translation to clinical use [191,192]. Peptidomimetics are *de novo* synthesized or modified peptide sequences with enhanced antibacterial potency and the lack of the disadvantages of the native peptides, which are currently in development [205,206,207]. Brilacidin is a defensin mimetic (defensins are antimicrobial peptides in vertebrates, which are predominantly found in neutrophil granulocytes; they have important roles in innate immunity [208]), which has broad-spectrum antibacterial activity [151,192,209]. This peptide affects the bacterial cell membrane in a similar fashion to daptomycin [151]. The main advantage of brilacidin is that it also exerts antimicrobial activity against the non-replicating, dormant forms (in case of *S. aureus*, small-colony variants) of bacteria [151,192,209]. Brilacidin is currently enrolled in Phase II. trials for the treatment of SSTIs and oral mucositis [192]. The therapeutic application of phages in the treatment of infectious diseases has been around since prior to the rise of antibiotics [200,201,210]. Due to the increase of MDR-infections, many people consider these bacteria-specific viruses to be potential therapeutic alternatives. There is an increasing number of case reports, where a seemingly untreatable infection (usually due to pan-resistant *Enterobacteriaceae*) was cured by the application of phages, therefore it would be valid to assume that they could also have significance in the treatment of MRSA infections. Although the regulatory environment is not yet defined, phages and artilysins (phage-derived lytic proteins) may be useful in MRSA-associated SSTIs. However, for their widespread use, an appropriate formulation for oral/parenteral use and the precise composition of effective phages/enzymes needs to be defined beforehand [200,201,210,211]. Novel drug targets include FabI inhibitors (FabI or enoyl-ACP-reductase is a key enzyme of unsaturated fatty acid synthesis in bacteria [212]), such as Debio 1452 and its prodrug Debio 1450 (or afabicin), which are in Phase II trials for the therapy of SSTIs that are caused by methicillin-susceptible and resistant *S. aureus* [11,213]. Many researchers are interested in the development of metal-based nanoparticles (NPs) with antimicrobial properties for use in MRSA infections [214]. Various NPs containing different metals (titanium, magnesium, gold, silver, zinc, copper, bismuth, etc.) showed promising activity against this pathogen, even though the penetration of *S. aureus* biofilms, while others (such as aluminum) were not recommended, based on experimental results. These studies predominantly administered these nanoparticles in some hydrogel form (i.e., for external use) or on the surface of an implanted medical device [215,216,217,218].

There were high hopes for the development of broad-spectrum, anti-MRSA/VRE carbapenems (e.g., tomopenem, razupenem), which bypassed the resistance that is caused by the modified PBPs and retaining their activity against Gram-negative organisms, however none of these agents thus passed onto clinical trials [219,220,221]. The closest thing in development with broad-spectrum activity is ceftaroline/avibactam, which is a combination of the anti-MRSA cephalosporin and a non-β-lactam-type β-lactamase-inhibitor [222,223].

New and emerging oligonucleotide-based antibacterial strategies, such as RNA-interference (RNAi), aptamers (single strand nucleic acids; ssRNA or ssDNA), and the use of CRISPR/Cas (clustered, regularly interspaced, short palindromic repeats, and CRISPR-associated protein 9, respectively) against MRSA and other MDR pathogens are also intensively studied, however, these are probably decades away from potential real-life applications [214,224,225]. The extremely high costs and risk-to-benefit ratio (which is especially high to antimicrobial research) is discouraging pharmaceutical companies to invest in studies targeting antibiotics, especially because, in addition to the physico-chemical characteristics, formulation, and adverse events of these compounds, companies have to keep in mind that microorganisms will develop resistance sooner or later, like in any facet of drug design and development [40,198,226,227,228]. Unfortunately, this is a general concern involving all MDR bacteria, not only MRSA, where clinicians are facing worsening odds to provide their patients with adequate therapy [35,80,81]. Additionally, some of these compounds are protein-based, therefore the potential for hypersensitivity and parenteral-only administration are additional hindering factors [203,229]. The development of novel antibiotics may be further complicated by their potential to affect the normal intestinal microbiota (i.e., their “ecological impact”). Drugs that kill the gut microbiota may select for *C. difficile*, VRE, ESBL-, and carbapenemase-producing *Enterobacteriaceae*, leading to additional issues for the patients in the future [230].

## 5. Concluding Remarks

*S. aureus* is a fine example of a successful pathogen, which was able to morph and adapt with such tenacity to the changing landscape of modern medical interventions (e.g., implanted devices, catheters) and the available antimicrobial agents. Penicillin-, methicillin-, (and to a lesser extent) vancomycin-resistant strains of staphylococci appeared one after another due to the selection pressure, proving that the antibiotic “arms race” is very real, and humanity as a whole is still on the losing side. MRSA infections emerged in nosocomial settings; however, in the 21^st^ century, the infections that were acquired in the community setting are a more pressing concern. MRSA is still an important factor of mortality (especially as a causative agent of endocarditis and bacteremia) all around the globe. Vigilant screening may reduce the number of patients where carriage turns into infection, while typing is useful in obtaining information pertaining to the local and global epidemiology, and the spread of successful clones. Alternative therapeutic modalities for MRSA are, in fact, being developed, though with no time-frame or guarantee that they will be successful in a clinical evaluation. One would argue that the current therapeutic armamentarium (and the number of drugs in development) could provide us with temporary safety; however, this hubris could also lead to the demise of the healthcare system, as we know it.

## Figures and Tables

**Table 1:** Summary of advantages and disadvantages for various drug classes involved in the treatment of MRSA infections [59,60,61,62,63,64,65,66,67,68,69,70,71,72,73,74,75,76,77,78,79,80,81,82,83,84,85,86,87,88,89,90,91,92,93,94,95,96,97,98,99,100,101,102,103,104,105,106,107,108,109,110,111,112,113,114,115,116,117,118,119,120,121,122,123,124,125,126,127,128,129,130,131,132,133,134,135,136,137,138,139,140,141,142,143,144,145,146,147,148,149,150,151,152,153,154,155,156,157,158,159,160,161,162,163,164,165,166,167,168,169,170,171,172,173,174,175,176,177,178,179,180,181,182,183,184,185].

Antibiotic Class (with Examples)	Advantages Indications (in Italics)	Disadvantages
SMX/TMP	Available for oral and parenteral use Good tolerability Price of therapy *Wide range of indications*	Resistance levels iv. infusion has to be administered in a large volume of fluid
Tetracyclines/Glycylcyclines (doxycycline, tygecycline)	Broad spectrum activity *Wide range of indications (tigecycline: SSTIs, cIAI, CAP)*	Doxycycline: resistance levels Tygecycline: black box warning, iv. only Severe nausea and vomiting (dose-limiting side effect)
Novel tetracycline-derivatives (*eravacycline, omadacycline*)	Broad spectrum activity *CAP, SSTIs*	Severe nausea and vomiting (dose-limiting side effect) Parenteral only Resistance expression/horizontally transmitted resistance genes
Glycopeptides (*vancomycin, teicoplainin*)	Gold standard of MRSA-therapy for a long time Extensive clinical data available regarding its usePrice of therapy *Wide range of indications*	MIC creep Parenteral only (with exceptions) TDM required (due to nephrotoxicity and ototoxicity) Resistance expression (hVISA, VISA, VRSA)
Lipoglycopeptides (*telavancin, dalbavancin, oritavancin*)	Long half-life (single-dose therapy) Useful in OPAT There is no need for TDM *SSTIs, bone and joint infections* *HAP, VAP (telavancin)*	Parenteral only Price of therapy Cannot be removed by dialysis Increased mortality in renal insufficiency Resistance expression/horizontally transmitted resistance genes
Oxazolidinones (*linezolid, tedizolid*)	Available for oral and parenteral use *SSTIs, bone and joint infections*	Drug-drug interactions MAO-inhibition (Serotonin-syndrome) Price of therapy Resistance expression/horizontally transmitted resistance genes
Lipopeptides (*daptomycin*)	*Bloodstream infections, infective endocarditis, SSTIs*	Not useful in pneumonia Parenteral only Resistance expression/horizontally transmitted resistance genes
5th generation cephalosporins (*ceftaroline, ceftobiprole*)	Good tolerability *SSTIs, CAP, HAP, MRSA bacteremia*	Price of therapy Hydrolized by ESBLs (mixed infections) Resistance expression/horizontally transmitted resistance genes
Older fluoroquinolones (*ciprofloxacin, levofloxacin, moxifloxacin*)	Available for oral and parenteral use Extensive clinical data available regarding their use Good tolerability Accumulation in the intracellular space Price of therapy Broad-spectrum activity *Wide range of indications*	Side effect profile (especially in light of recent developments) Resistance levels and rapid resistance development
Next-generation fluoroquinolones (*delafloxacin; avarofloxacin, finafloxacin, zaborfloxacin, nemonoxacin*)	Available for oral and parenteral use Broad-spectrum activity Accumulation in the intracellular space *Presently studied in a wide range of indications (e.g., cSSTI, CAP, HAP, cUTI* *MDR gonorrhea)*	*Black box warining* Side effect profile Price of therapy
*Mupirocin*	Price of therapy Dose-dependent bactericidal activity *Topical agent for MRSA nasal decolonization* *Additonal indications are being studied*	Resistance development Risk of toxicity when used orally/parenterally

## Abbreviations

**Abbreviations**	**Full**
**AST**	antimicrobial susceptibility testing
**CA**	community-acquired; CAMERA
**CAMERA**	combination antibiotic therapy for methicillin-resistant *Staphylococcus aureus* infection (clinical trial)
**CAP**	community-acquired pneumonia
**CC**	clonal complex
**CDC**	US Centers for Disease Control and Prevention
**cgMLST**	core genome multi-locus sequence typing
**CLSI**	Clinical and Laboratory Standards Institute
**CoNS**	coagulase-negative *Staphylococcus*
**CO-MRSA**	community-onset MRSA
**CRISPR/Cas9**	lustered regularly interspaced short palindromic repeats/CRISPR associated protein 9
**CYP**	cytochrome P450
**DHFR**	dihydrofolate-reductase
**ESBL**	extended-spectrum β-lactamase
**ESCMID**	European Society of Clinical Microbiology and Infectious Diseases
**EU**	European Union
**EUCAST**	European Committee on Antimicrobial Susceptibility Testing
**FDA**	US Food and Drug Administration
**HA**	hospital-associated
**HAP**	hospital-acquired pneumonia
**hVISA**	heterogeneous vancomycin-intermediate *S. aureus*
**HLA**	human leukocyte antigen
**IAI**	intra-abdominal infection
**LA**	livestock-associated
**MALDI-TOF MS**	matrix-assisted laser desorption/ionization time-of-flight mass spectrometry
**MAO-A**	monoamine-oxidase-A
**MFS**	major facilitator superfamily
**MDR**	multidrug-resistant
**MIC**	minimal inhibitory concentration
**MLST**	multi-locus sequence typing
**MRSA**	methicillin/oxacillin-resistant *S. aureus*
**NGS**	next-generation sequencing
**NP**	nanoparticle
**OPAT**	outpatient parenteral antibiotic therapy
**PFGE**	pulse-field gel electrophoresis
**PBP**	penicillin-binding protein
**QS**	quorum sensing
**UTI**	urinary tract infection
**PDI**	prosthetic device infection
**PCR**	polymerase chain reaction
**PVL**	Panton–Valentine leucocidin
**QRDR**	quinolone resistance-determining region
**QTc**	corrected QT-interval
**RNA**	ribonucleic acid
**RNAi**	RNA-interference
**SCV**	small-colony variant
**SMX/TMP**	co-trimoxazole
**SSTI**	skin and soft tissue infection
**ST**	sequence type
**ssDNA**	single-strand DNA
**ssRNA**	single-strand RNA
**TDM**	therapeutic drug monitoring
**TAT**	turnaround time
**TLR**	toll-like receptor
**TSS**	toxic shock syndrome
**TSST**	toxic shock syndrome toxin
**UTI**	urinary tract infection
**VAP**	ventilator-associated pneumonia
**VISA**	vancomycin-intermediate *S. aureus*
**VNTR**	variable number tandem repeat
**VRE**	vancomycin-resistant enterococci
**VRSA**	vancomycin-resistant *S. aureus*
**WGS**	whole-genome sequencing
